# The Scottish Intercollegiate Guidelines Network (SIGN) 157: Guidelines on Risk Reduction and Management of Delirium

**DOI:** 10.3390/medicina55080491

**Published:** 2019-08-15

**Authors:** Roy L. Soiza, Phyo K. Myint

**Affiliations:** Ageing Clinical and Experimental Research Group (ACER), Rm 1:128, Polwarth Building, University of Aberdeen, Aberdeen AB25 2ZN, UK

**Keywords:** acute confusion, ageing, delirium, diagnosis, encephalopathy, guideline, treatment

## Abstract

The Scottish Intercollegiate Guidelines Network (SIGN) guideline on delirium is a major advance on existing guidelines on this condition. This is particularly important given the evidence it is frequently under-diagnosed and inadequately managed despite being common and frequently associated with significant patient and carer distress and poor outcomes. The guidelines recommend using the 4A’s test to help detect delirium. A bundle of mostly non-pharmacological therapies minimise the risk of developing delirium and can help those who develop the condition. The importance of medical optimisation by an experienced professional in those at risk of delirium is highlighted with new recommendations for people in intensive care and surgical settings. There is guidance on follow-up of people with delirium, which should become routine. This commentary piece focusses on areas with the greatest potential to improve the experience and outcomes of those with delirium, and briefly discusses areas of ongoing uncertainty.

## 1. Background

Delirium is a state of acute deterioration in mental functioning that occurs soon after acute medical illness, surgery, trauma, or a change in medications [1]. It causes significant patient and caregiver distress [2] and is associated with multiple serious complications, i.e., falling and/or worsened outcomes that increase the length of stay in hospitals and have higher rates of death and institutionalization [1]. Despite this, delirium is frequently underdiagnosed and poorly managed in clinical practice [3,4]. A systematic appraisal of existing guidelines revealed significant deficiencies in a wide range of quality measures, including applicability, editorial independence, currency, and visibility [5]. The development of a robust new guideline by the Scottish Intercollegiate Guidance Network (SIGN) is therefore a very welcome development [6]. It showcases the state-of-the-art practices in delirium prevention and management, providing a list of 12 evidence-based recommendations (see Section 1.1) and expert opinion-based ‘good practice points’ (see Section 1.2), which are applicable to a wide range of healthcare settings. In this article, we discuss the recommendations that have the greatest potential to improve the experience and outcomes of those with delirium, while briefly highlighting areas of continuing uncertainty.

### 1.1. List of All Recommendations in the Scottish Intercollegiate Guidance Network (SIGN) Guideline on Risk Reduction and Management of Delirium

Recommendations:

- The 4AT tool should be used for identifying patients with probable delirium in emergency department and acute hospital settings.
- Use of the 4AT tool could be considered for use in community or other settings for identifying patients with probable delirium.
- For intensive care unit settings, Confusion Assessment Method – Intensive Care Unit (CAM-ICU) or Intensive Care Delirium Screening Checklist (ICDSC) should be considered to help identify patients with probable delirium.
- Computed tomography (CT) brain scan should not be used routinely but should be considered in hospital patients with delirium in the presence of:
  ○ new focal neurological signs;
  ○ a reduced level of consciousness (not adequately explained by another cause);
  ○ a history of recent falls;
  ○ a head injury (patients of any age);
  ○ anticoagulation therapy.
- Electroencephalogram should be considered when there is a suspicion of epileptic activity or non-convulsive status epilepticus as a cause of a patient’s delirium.
- The following components should be considered as part of a package of care for patients at risk of developing delirium:
  ○ orientation and ensuring patients have their glasses and hearing aids;
  ○ promoting sleep hygiene;
  ○ early mobilization;
  ○ pain control;
  ○ prevention, early identification, and treatment of postoperative complications;
  ○ maintaining optimal hydration and nutrition;
  ○ regulation of bladder and bowel function;
  ○ provision of supplementary oxygen, if appropriate.
- With the aim of avoiding excessively deep anesthesia, depth of anesthesia should be monitored in all patients aged over 60 years who are under general anesthesia during surgery expected to last for more than one hour.
- The use of earplugs should be considered as part of a sleep-promotion strategy in intensive care.
- All patients at risk of delirium should have a medication review conducted by an experienced healthcare professional.
- Healthcare professionals should follow established pathways of good care to manage patients with delirium.
  ○ First, consider acute, life-threatening causes of delirium, including low oxygen level, low blood pressure, low glucose level, and drug intoxication or withdrawal.
  ○ Systematically identify and treat potential causes (medications, acute illness, etc), noting that multiple causes are common.
  ○ Optimize physiology, management of concurrent conditions, environment (reduce noise), medications, and natural sleep, to promote brain recovery.
  ○ Specifically detect, assess causes of, and treat agitation and/or distress, using nonpharmacological means only if possible (see Section 3 for pharmacological treatment).
  ○ Communicate the diagnosis to patients and caregivers, encourage involvement of caregivers, and provide ongoing engagement and support.
  ○ Aim to prevent complications of delirium such as immobility, falls, pressure sores, dehydration, malnourishment, and isolation.
  ○ Monitor for recovery and consider specialist referral if not recovering.
  ○ Consider follow-up
- Healthcare professionals should be aware that older people may have pre-existing cognitive impairment that may have been undetected or exacerbated in the context of delirium. Appropriate cognitive and functional assessment should be considered. Timing of this assessment must take into account persistent delirium.

### 1.2. List of All ‘Good Practice Points’ in the SIGN Guideline on Risk Reduction and Management of Delirium.

Good Practice Points:

- A formal assessment and diagnosis must be made by a suitably trained clinician whenever patients with probable delirium are identified.
- Where delirium is detected, patients and their family/caregivers should be informed of the diagnosis.
- Where delirium is detected, the diagnosis of delirium should be clearly documented to aid transfers of care (e.g., handover notes, referral and discharge letters).
- Consideration should be given to imaging patients with non-resolving delirium where no clear cause is identified or there are features to suggest primary central nervous system pathology.
- Lumbar puncture should not be performed routinely on patients with delirium.
- Ward moves should be avoided wherever possible for patients at risk of delirium.
- Prior to surgery, patients and caregivers should be advised of the risk of developing delirium, to alleviate distress and help with management if it does occur.
- Where possible, assistance should be sought from a patient’s relatives and caregivers to deliver care to reduce the risk of delirium developing.
- Areas with patients at high-risk of delirium, such as trauma orthopaedic wards, should have protocols for commonly required medication (e.g., analgesia and anti-emesis) that contain choices for first-line treatments that minimise the risk of causing delirium.
- Promote cognitive engagement, mobilization, and other rehabilitation strategies.
- Patient records should be coded to highlight a previous episode of delirium so that hospital staff are aware of the increased risk on readmission.
- Ensure that delirium is noted in the discharge letter for the primary care team.
- All patients who have had delirium should be reviewed by the primary care team.
- In patients who have experienced delirium in intensive care units (ICU), consideration should be given to follow-up for psychological sequelae including cognitive impairment.

## 2. Detection of Delirium

The SIGN guideline specifically recommends routinely using the 4AT tool [7] in emergency department and acute hospital settings and either the CAM-ICU or ICDSC [8] in ICU. There was insufficient evidence to make any firm recommendations in other settings, such as primary care, but the guideline suggests the 4AT could be used to identify those with probable delirium. The 4AT was the preferred tool due to its brevity, simplicity, applicability, and predictive properties. No specific staff training is required for its use and numerous independent validation studies have found high patient completion rates and sensitivity (86–100%) and specificity (65–82%) that compares favorably to other tools. It is important to note that most tools are essentially screening tools (e.g., 4AT, ICDSC) with higher sensitivity than specificity. Despite being a commonly used tool and closer to having ‘diagnostic’ properties, the CAM is less favored in the guideline. This was principally due to its requirement for training and concerns that it lacks sensitivity in less experienced hands. The guideline states that it is important to follow any positive assessments with additional assessment by a suitably trained clinician against International Classification of Diseases (ICD-10) or Diagnostic and Statistical Manual (DSM-5) criteria. It also states that a negative result does not rule out delirium completely, as delirium classically fluctuates or can occur after admission. 

Although this is helpful and should minimize under-diagnosis of delirium, some questions remain. Should all admissions to hospital be screened or should detection tools be targeted, for example to those aged over 65? How should we monitor for incident delirium? In what patient groups and how frequently? Do available tools have utility in community and long-term care settings? There is currently insufficient evidence to answer these questions.

## 3. Managing Delirium

Reinforced throughout the SIGN guideline is the importance of making the diagnosis of delirium and communicating it to the patient, their relatives, and all relevant healthcare professionals involved in their care. This includes clearly documenting the diagnosis in the clinical record and any transfer and discharge documentation. There are helpful sections on communication and patient education, with resources and checklists of important points to consider helping patients and relatives become better informed and more actively involved in their condition and treatment. This is especially pertinent where individuals lose their capacity to consent to investigation and treatment due to delirium.

Moreover, the SIGN guidelines acknowledge that the evidence-base for effective treatments in delirium is weak. Nevertheless, it recommends following an established, multi-component pathway of interventions to help identify underlying causes, since delirium is usually multi-factorial. Though no specific pathway is recommended, a couple of potentially suitable examples (the TIME bundle and the Scottish Delirium Association pathway [9]) are included as appendices. There is a wealth of information on appropriate clinical investigations to look for underlying causes, but interestingly some of the focus of the recommendations are on the futility of some over-used tests. For example, CT scans of the head and lumbar puncture should not be used routinely due to low diagnostic yield, though they are still recommended in some specific situations, for example where there are localizing signs on neurological examination to suggest a brain lesion. Conversely, the guidelines acknowledge recent evidence suggesting epileptic activity and non-convulsive status in delirium is higher than it is widely appreciated. Though it does not recommend its routine use, the guidelines recommend that electroencephalography (EEG) should be undertaken when there is clinical suspicion of epilepsy or non-convulsive status as a cause of delirium. This may also include its use in unexplained, non-resolving delirium.

The guidelines do not recommend routine use of any pharmacological treatment of delirium and particularly highlight a recent Cochrane review finding that antipsychotics did not reduce delirium severity, duration, or alter mortality [10] in non-ICU settings. Evidence of their efficacy in the ICU was insufficient despite some limited evidence they might reduce duration of delirium [11]. There was insufficient evidence to recommend use of dexmedetomidine, acetylcholinesterase inhibitors, or benzodiazepines. However, use of urgent pharmacological intervention is advocated in patients with ‘intractable distress’ or where the safety of the patient or others is compromised.

Finally, the guidelines make clear recommendations that all patients with delirium should be followed up in primary care to check for common sequela, including depression, post-traumatic stress disorder and enduring cognitive impairment that may lead to a diagnosis of dementia.

## 4. Risk Reduction and Prevention of Delirium

Since treatments for delirium are of limited proven efficacy, many of the guideline recommendations focus on preventing delirium in those at high-risk of developing it. A multi-component non-pharmacological intervention promoting early mobilization, regular orientation, minimizing sensory impairment, and optimizing hydration and nutrition significantly reduces incident delirium rates by almost a third, but only in those at higher risk [12,13]. The definition of a ‘high-risk’ population is undefined in the guideline due to lack of consensus but, typically, studies showing efficacy were undertaken in older people (usually defined as over 65 years old), those with pre-existing cognitive impairment, or adults of any age in ICU. There are a number of specific recommendations for patients in ICU, such as provision of hearing plugs to minimize sleep disturbance, and for those undergoing major surgery, such as minimizing depth of anesthesia through intra-operative monitoring. Recommendations around pharmacological risk reduction and prevention are focuses on avoiding or minimizing doses of medications known to induce or exacerbate delirium, such as benzodiazepines, opioids, and anticholinergics. All patients at risk of delirium should have their prescriptions optimized by an experienced professional. Clinical departments with high prevalence of delirium are encouraged to develop protocols for commonly required medications (e.g., analgesia) that contain treatments that minimize the risk of causing delirium. As for the treatment of delirium, no specific pharmacological therapies to prevent delirium are recommended, including some that are routinely used in some clinical areas, such as antipsychotics and melatonin. Although an early draft of the guideline suggested peri-operative dexmedetomidine could be considered in high-risk patients, the final version of the guideline concludes the evidence for its efficacy is not good enough to warrant any recommendation. This decision centered around concerns that trials showing benefit were largely limited to those where the control group had received higher doses of benzodiazepines and other sedatives that are known to cause delirium.

## 5. Comparison between SIGN and National Institute of Healthcare and Care Excellence (NICE) Guidelines

Although the new guideline is broadly similar to the National Institute of Healthcare and Care Excellence (NICE CG 103, 2010) guidelines on delirium, there are some important differences. SIGN is more specific about how best to detect delirium, particularly in non-ICU settings. The recommendations for preventing delirium are essentially the same, though the updated SIGN guideline includes a stronger evidence base for multi-component interventions from recent studies. The new guidelines on investigating and treating delirium are much more detailed than that in NICE, with a stronger emphasis on non-pharmacological interventions and encouraging the active participation of family and caregivers. Conversely, the recommendations around pharmacological treatment are more reserved, with no specific recommendation for haloperidol or any other agent due to the lack of evidence of efficacy. Recommendations on follow-up are also included only in the SIGN guidelines.

## 6. Implementing the Guideline and the Future of Delirium Care

Guidelines have potential to do harm [14], especially if they are applied indiscriminately or used as a substitute for clinical reasoning. However, the SIGN guideline reinforces throughout the need for person-centered care and judicious application of all recommendations according to the needs of the individual patient. It includes recommendations on implementation and quality assurance for Scottish health boards that could easily be adopted elsewhere. It helpfully lists opportunities for future research on important clinical questions that remain unanswered, such as the impact of follow-up clinics on the mental health of people who have experienced delirium. It promises to be a landmark publication to help bridge the gap between desired and actual clinical practice [15]. Widespread implementation of its recommendations would have a major beneficial impact on the prevalence of delirium and the poor outcomes and experiences for those with the condition.
